# MIEF2 over-expression promotes tumor growth and metastasis through reprogramming of glucose metabolism in ovarian cancer

**DOI:** 10.1186/s13046-020-01802-9

**Published:** 2020-12-14

**Authors:** Shuhua Zhao, Xiaohong Zhang, Yuan Shi, Lu Cheng, Tingting Song, Bing Wu, Jia Li, Hong Yang

**Affiliations:** 1 Department of Gynaecology and Obstetrics, Xijing Hospital, Fourth Military Medical University, 15 Changle Western Road, Xi’an, 710032 Shaanxi China; 2 Department of Geriatrics, the 940th Hospital of Joint Logistics Support Force of Chinese People’s Liberation Army, Lanzhou, China

**Keywords:** Mitochondrial elongation factor 2, Growth, Metastasis, Glycolysis, OC

## Abstract

**Background:**

Increasing evidence has revealed the close link between mitochondrial dynamic dysfunction and cancer. MIEF2 (mitochondrial elongation factor 2) is mitochondrial outer membrane protein that functions in the regulation of mitochondrial fission. However, the expression, clinical significance and biological functions of MIEF2 are still largely unclear in human cancers, especially in ovarian cancer (OC).

**Methods:**

The expression and clinical significance of MIEF2 were determined by qRT-PCR, western blot and immunohistochemistry analyses in tissues and cell lines of OC. The biological functions of MIEF2 in OC were determined by in vitro and in vivo cell growth and metastasis assays. Furthermore, the effect of MIEF2 on metabolic reprogramming of OC was determined by metabolomics and glucose metabolism analyses.

**Results:**

MIEF2 expression was significantly increased in OC mainly due to the down-regulation of miR-424-5p, which predicts poor survival for patients with OC. Knockdown of MIEF2 significantly suppressed OC cell growth and metastasis both in vitro and in vivo by inhibiting G1-S cell transition, epithelial-to-mesenchymal transition (EMT) and inducing cell apoptosis, while forced expression of MIEF2 had the opposite effects. Mechanistically, mitochondrial fragmentation-suppressed cristae formation and thus glucose metabolism switch from oxidative phosphorylation to glycolysis was found to be involved in the promotion of growth and metastasis by MIEF2 in OC cells.

**Conclusions:**

MIEF2 plays a critical role in the progression of OC and may serve as a valuable prognostic biomarker and therapeutic target in the treatment of this malignancy.

**Supplementary Information:**

The online version contains supplementary material available at 10.1186/s13046-020-01802-9.

## Background

Ovarian cancer (OC) is one of the most common gynecological malignancies in women worldwide [1]. Despite advances of combined therapies including surgery and adjuvant approaches, the prognosis of patients with ovarian cancer continues to be poor. The molecular mechanisms involved in ovarian carcinogenesis are still poorly defined, which limited the effective methods for clinical treatment [2, 3]. Accordingly, identification of novel molecular alterations contribute to the metastatic growth of OC is critical for development of novel diagnostic and therapeutic strategies to obtain more effective treatment in this malignancy.

Alteration of glucose metabolism characterized by increased aerobic glycolysis (also known as Warburg effect) has been well-established as one of the hallmarks of cancer [4], which contributes tumor growth and metastasis by providing not only energy but also substrates for biosynthesis [5–7]. Emerging studies has revealed mitochondrial dysfunction as one of the most common reasons for increased aerobic glycolysis in cancer cells [8–11], suggesting that identification of novel regulators contributing mitochondrial dysfunction may uncover molecular mechanisms underlying the increased aerobic glycolysis in cancer cells.

Mitochondria are crucial organelles involved in cellular metabolism regulation. The morphology of mitochondrial is continuously remodeled by the balance between fission and fusion events [12–14]. During recent years, the close links between fragmented mitochondrial networks and cancer have been revealed in various types of human cancers [15], including liver [16, 17], breast [18, 19], lung [20, 21], colon [22] and ovarian [23, 24] cancers. In addition, abnormal expressions of mitochondria fission and fusion proteins such as DRP1 (dynamin-related protein 1) and MFN1 (mitofusion 1) have also been observed. MIEF2 (mitochondrial elongation factor 2) is a mitochondrial outer membrane protein that functions in the regulation of mitochondrial fission [25]. However, the expression, clinical significance and biological functions of MIEF2 are still largely unclear in human cancers, including ovarian cancer (OC).

In this study, we conduct the first study on MIEF2 in ovarian cancer to clarify its expression pattern, clinical significance, biological effects in this malignancy.

## Methods

### Cell culture and tissue collection

Human ovarian cancer (OC) cell lines (A2780, SKOV3, OVCAR3, HEY and ES2) and an immortalized but non-tumorigenic ovarian epithelial cell line IOSE80 were purchased from the Cell Bank of Chinese Academy of Sciences (Shanghai, China). All cell lines were authenticated by STR profiling test to confirm their identities and cultured in Dulbecco’s Modified Eagle (DMEM) or RPMI-1640 Medium supplied with 10% fetal bovine serum, 100 U/ml penicillin and streptomycin in an incubator with 5% CO_2_ at 37 °C. In addition, 152-paired tumor and surrounding non-tumor tissue samples (30 for qRT-PCR analysis; 122 for IHC staining analysis) were collected from ovarian cancer patients at the First Affiliated Hospital of the Fourth Military Medical University in Xi’an, China. The study was approved by the Ethics Committee of Fourth Military Medical University. Informed consent was obtained from all individual participants included in the study.

### Over-expression and knockdown of target genes

The transient knockdown of MIEF2 was obtained by transfection of small interference RNA (siRNA) with Lipofectamine 2000 (Invitrogen, California, USA) according to the manufacturer’s protocol. The sequence of si-MIEF2#1 was 5′- ACACCTAAGTTCAGCACTATAGCAC-3′; The sequence of si-MIEF2#2 was 5′- GCCATGCCTTGAAGATGTGAATAAA-3′. The stable knockdown of MIEF2 was obtained by transfection with the shRNA expression vector generated by a pSilencer™ 3.1-H1 puro vector (Ambion, Austin, TX, USA). For MIEF2 over-expression, the open reading frame sequence of MIEF2 was amplified and cloned into a pcDNA™3.1(C) vector (Invitrogen, V790–20). Synthetic miRNA mimics and control oligonucleotide (NC) were purchased from RiboBio Inc. (Guangzhou, China) and transfected into OC cells with Lipofectamine 2000 according to the manufacturer’s instruction.

### RNA extraction and quantitative real-time PCR (qRT-PCR)

RNA was extracted from OC cells using Trizol reagent (Invitrogen, USA). Then, reverse transcription of extracted RNA was performed using a PrimeScript® RT reagent kit (Takara, Japan) following to the manufacturer’s instructions. PCR amplification was performed using SYBR Premix Ex Taq (Takara, Japan). Relative expressions of target genes were calculated using the 2^-△△Ct^ method and β-actin was considered as a reference gene for normalization. The primer sequences were listed in the supplementary Table 1.

### Western blot analysis

Cells were lysed with RIPA buffer containing the protease inhibitor cocktail (Sigma, USA). Proteins (35 μg) were separated in SDS-PAGE gels and transferred onto PVDF membrane (Millipore, USA). The membranes were then blocked with 5% milk and probed with primary and secondary horseradish-peroxidase-labeled antibodies. After washing three times, the signaling was detected by an enhanced chemiluminescence detection system (ECL; Amersham Pharmacia Biotech). Primary antibodies used in the present study were listed in the supplementary Table 2.

### Immunohistochemistry (IHC) analysis

Paraffin-embedded tissue sections (4 μM) were rehydrated, blocked with 3% hydrogen peroxide and treated with hot citrate buffer. After that, primary antibodies of MIEF2 and Ki-67 were added and incubated overnight at 4 °C. The results were determined by an IHC detection kit (MXB, Fuzhou, China) according to the manufacturer’s protocol. The staining intensity was scored independently by two observers. Briefly, the scores for the proportion of positive staining (1, < 5%; 2, 5–30%; 3, 30–70%; 4, > 70%) and staining intensity (0, no staining; 1, weak; 2, moderate; 3, strong) were multiplied for each observer and then averaged.

### MTS assay

A total of 1 × 10^3^ OC cells were plated into 96-well cell culture plates (020096, Xinyou Biotech, Hangzhou, China). After grown for 0, 24, 48, 72, 96 and 120 h, 20 μL MTS solution (Promega, G3581) was added to each well and incubated 2 h at 37 °C. Finally, the absorption values at 490 nm were measured with a Bio-Rad’s microplate reader to determine the relative cellular proliferation capacities.

### Colony formation assay

A total of 500 OC cells were seeded into 6-well plates and cultured for 15 days. Formed colonies were fixed with 4% paraformaldehyde and stained with crystal violet for 15 min, respectively.

### Flow cytometry analysis of cell cycle and cell apoptosis

OC cells were washed with PBS and then analyzed with a cell cycle (F-6012, US Everbright Inc) kit or an Annexin V (FITC-conjugated) apoptosis (F-6012, US Everbright Inc) kit, according to their manufacturer’s protocols. Cell cycle distribution in each phase and percentage of apoptotic cells were determined with a flow cytometry (Beckman, Fullerton, CA).

### Wound-healing cell migration assay

OC cells were cultured in 6-well plates and grown to 90% confluence. Then, a plastic pipette tip was used for scratching in the bottom of the wells. After washing two times with the culture medium without fetal bovine serum, images were captured with a light Olympus microscope at 0 and 24 h. Image J software was used for the determination of relative migration in each group.

### Matrigel invasion assay

Matrigel-coated Invasion Chamber (BD Biosciences, UJ, USA) was used for assessment of cell invasion. Briefly, 2 × 10^4^ OC cells were seeded in the upper chamber of the transwell insert in serum-free culture medium and cultured for 48 h. Penetrated cells were fixed with 4% paraformaldehyde and stained with 0.5% crystal violet. The number of penetrated cells in each group was counted under a light Olympus microscope.

### In vivo tumorigenicity assay

A total of 1 × 10^7^ OC cells with different MIEF2 level were subcutaneously injected into the flank of 4–5 weeks old female BALB/c athymic nude mice (six mice per group). Tumor volumes were measured once every week for 5 weeks. Then, tumors were removed and their sizes and weights were determined. The animal study was approved by the Institutional Animal Experiment Committee of Xijing hospital and carried out in accordance with the UK Animals (Scientific Procedures) Act, 1986.

### In vivo metastatic assay

A total of 5× 10^6^ OC cells with different MIEF2 level were intravenously injected into the tail vein of 4–5 weeks old female BALB/c athymic nude mice. After 7 weeks, the mice were sacrificed and metastatic tumors formed in their lungs were determined with hematoxylin and eosin (H&E) staining analysis.

### Detection of oxygen consumption rate (OCR) and mitochondrial respiratory chain complexes activities

OC cells were plated into an XF96 plate at a density of 1.0 × 10^4^ cells/well and cultured overnight. The XF96 Extracellular Flux Analyzer (Seahorse Bioscience) was used for detection of cellular oxygen consumption in OC cells, according to the manufacturer’s protocol.

A commercial kit from abcam (ab110419) was used for detection of the activities of the five mitochondrial respiratory chain complexes, following to the manufacturer’s protocol. The absorption values at 340 nm (complexes I and V), 550 nm (complexes III and IV) and 600 nm (complex II) were measured with a Bio-Rad’s microplate reader.

### Detection of ATP

ATP production was determined with ATP Determination Kit (Thermo Fisher Scientific, A22066) following to the manufacturer’s instruction. Briefly, OC cells with different treatment were homogenized in the lysis buffer. The results were determined by luminescence (Promega, Glomax 20/20 luminometer) and normalized to protein concentration.

### Determination of glucose consumption and lactate production

Glucose and lactate detection kits purchased from Nanjing jiancheng Bioengineering institute (Nanjing, China) were used for determination of glucose and lactate concentrations before and after 24 h cell culture in OC cells, following to their manufacturer’s protocols. In addition, a pH meter (PB-11 Basic Meter, The Netherlands) was used for the measurement of pH value in cell culture medium.

### Statistical analysis

Results were presented as mean ± SEM. The SPSS software (17.0 version) was used for statistical analysis and *p* < 0.05 was considered as statistically significant (*). The two-tailed student’s t-test and one-way ANOVA with Tukey’s post-hoc test were used for comparisons between two or multiple groups, respectively. Kaplan-Meier method and log-rank test were used for overall and recurrence-free survival analyses.

## Results

### MIEF2 expression is increased in OC tissues and cell lines and associated with poor prognosis in patients with OC

**Fig. 1:**
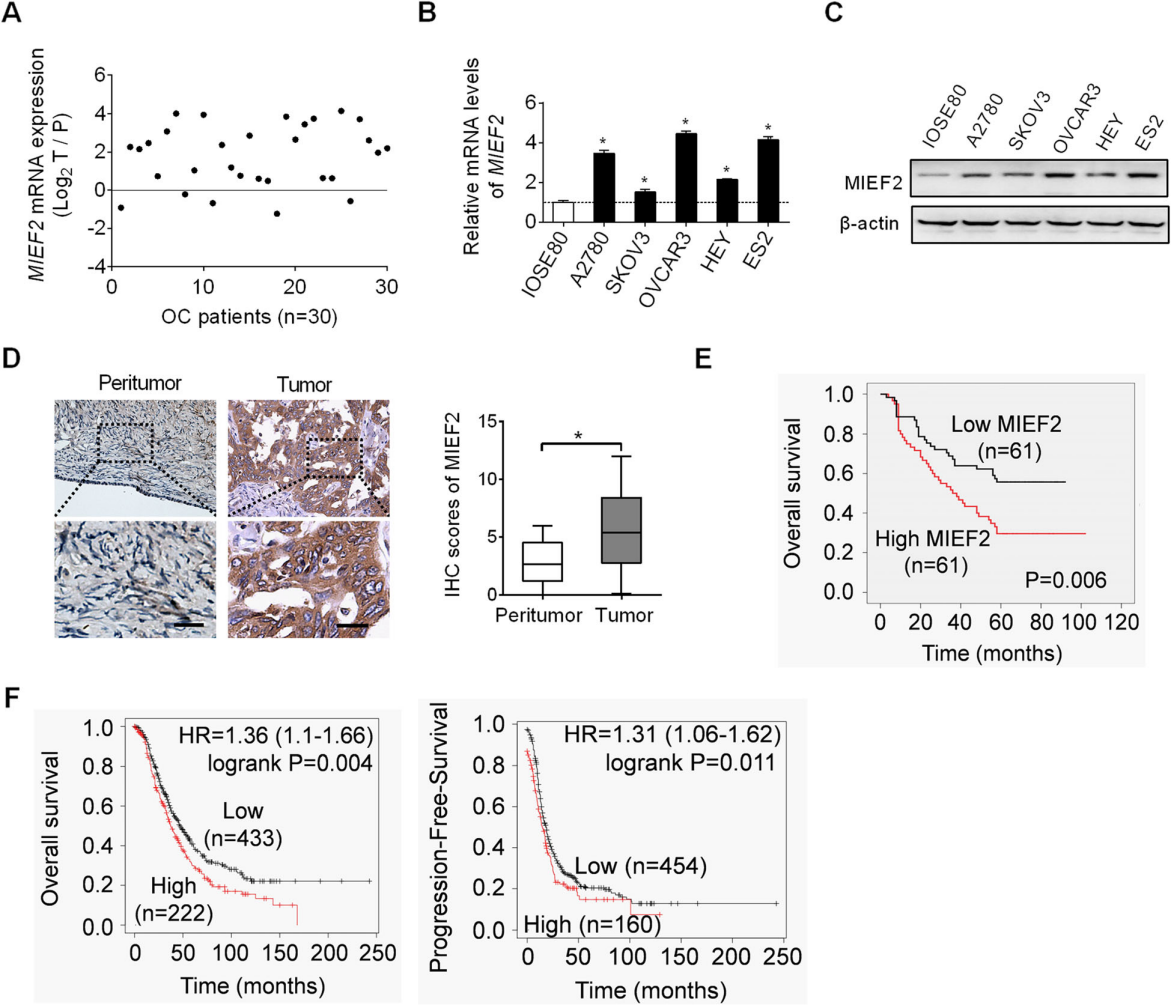
MIEF2 expression is increased in OC tissues and cell lines and associated with poor prognosis in patients with OC. **a** The expression of MIEF2 was evaluated by qRT-PCR analysis in paired tumor and peritumor tissues from 30 OC patients. (T, tumor; P, peritumor). **b** and **c** qRT-PCR and Western blot analysis of MIEF2 expression in five human OC cell lines (A2780, SKOV3, OVCAR3, HEY and ES2) and an immortalized ovarian epithelial cell line IOSE80. **d** Immunohistochemical (IHC) staining of MIEF2 in 122-paired OC tumor and peritumor tissues. Scale bar, 50 μm. **e** and **f** Bioinformatics analysis using the KM plotter was applied for overall (**e**) and progression-free (**f**) survival of MIEF2 in OC patients. *P < 0.05

The expression of MIEF2 was firstly evaluated in tumor and corresponding peritumor tissues from 30 patients with ovarian cancer (OC) using quantitative real-time PCR (qRT-PCR) analysis. Our results showed a significantly up-regulation of MIEF2 in OC tissues when compared with peritumor tissues (Fig. 1a). Consistently, increased MIEF2 expression was also detected in five OC cell lines (A2780, SKOV3, OVCAR3, HEY and ES2) as compared with an immortalized ovarian epithelial cell line IOSE80 (Fig. 1b-c).

To explore the relationship between MIEF2 expression and survival of OC patients, immunohistochemical (IHC) analysis was applied for detection of MIEF2 protein expression level in another 122-paired OC tumor and peritumor tissues. MIEF2 was significantly higher in tumor tissues of OC compared to peritumor tissues (Fig. 1d). Kaplan-Meier survival analysis demonstrated that OC patients with higher MIEF2 expression had obvious poorer overall survival as compared to MIEF2 lower expression patients (Fig. 1e). Consistent with this, bioinformatics analysis using the KM-plotter [26] also indicated that OC patients with high MIEF2 expression had significant shorter overall (*P* = 0.001) and progression free survival (Fig. 1f). Taken together, MIEF2 expression was up-regulated in OC tissues/cells and associated with poor prognosis for patients with OC.

### Knockdown of MIEF2 suppressed OC cell growth through induction of G1-S cell cycle arrest and cell apoptosis

**Fig. 2:**
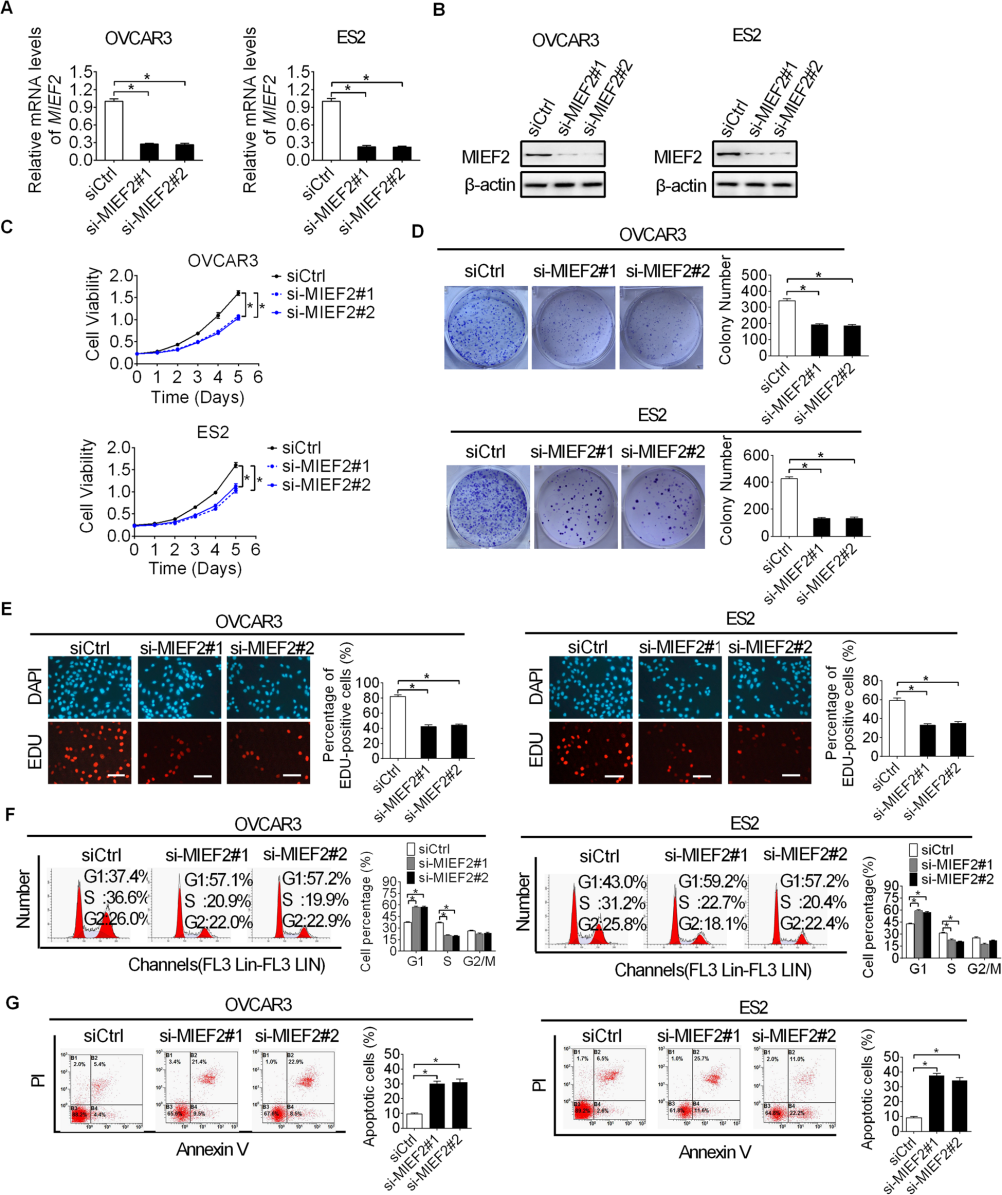
Knockdown of MIEF2 suppressed OC cell growth through induction of G1-S cell cycle arrest and cell apoptosis. **a** and **b** Knockdown of MIEF2 was confirmed by qRT-PCR and Western blot analysis in OVCAR3 and ES2 cells. siMIEF2, siRNA against MIEF2; siCtrl, control siRNA. **c** and **d** MTS cell viability and colony formation assays in OVCAR3 and ES2 cells with or without MIEF2 knockdown. **e** EdU incorporation assay was performed in OVCAR3 and ES2 cells with or without MIEF2 knockdown. Scale bars, 50 μm. **f** and **g** Flow cytometry analysis for cell cycle distribution and apoptosis in OVCAR3 and ES2 cells with or without MIEF2 knockdown. *P < 0.05

Increased MIEF2 expression implies that MIEF2 may function as an oncogene in the tumorigenesis of OC. To prove this, MIEF2 expression was knocked-down in OVCAR3 and ES2 cells (Fig. 2a and b) with relatively high MIEF2 expression as shown in Fig. 1b and Fig. 1c. Knockdown of MIEF2 significantly suppressed cell proliferation and colony formation in OVCAR3 and ES2 cells (Fig. 2c and d), as determined by MTS cell viability and colony formation assays. To characterize the mechanism by which MIEF2 knockdown suppressed OC cell growth, the effects of MIEF2 knockdown on cell proliferation and apoptosis were determined by EdU (5-ethynyl-2′-deoxyuridine) incorporation assay, as well as flow cytometry cell cycle distribution and apoptosis assays. As shown in Fig. 2e-g, knockdown of MIEF2 in OVCAR3 and ES2 cells resulted in significant lower percentage of proliferating cells (Fig. 2e) and cell cycle arrest at G1 phase (Fig. 2f), while a significant increase of cell apoptosis (Fig. 2g), suggesting that MIEF2 knockdown suppressed OC cell growth through induction of G1-S cell cycle arrest and cell apoptosis.

### MIEF2 knockdown suppressed migration and invasion of OC cells

**Fig. 3:**
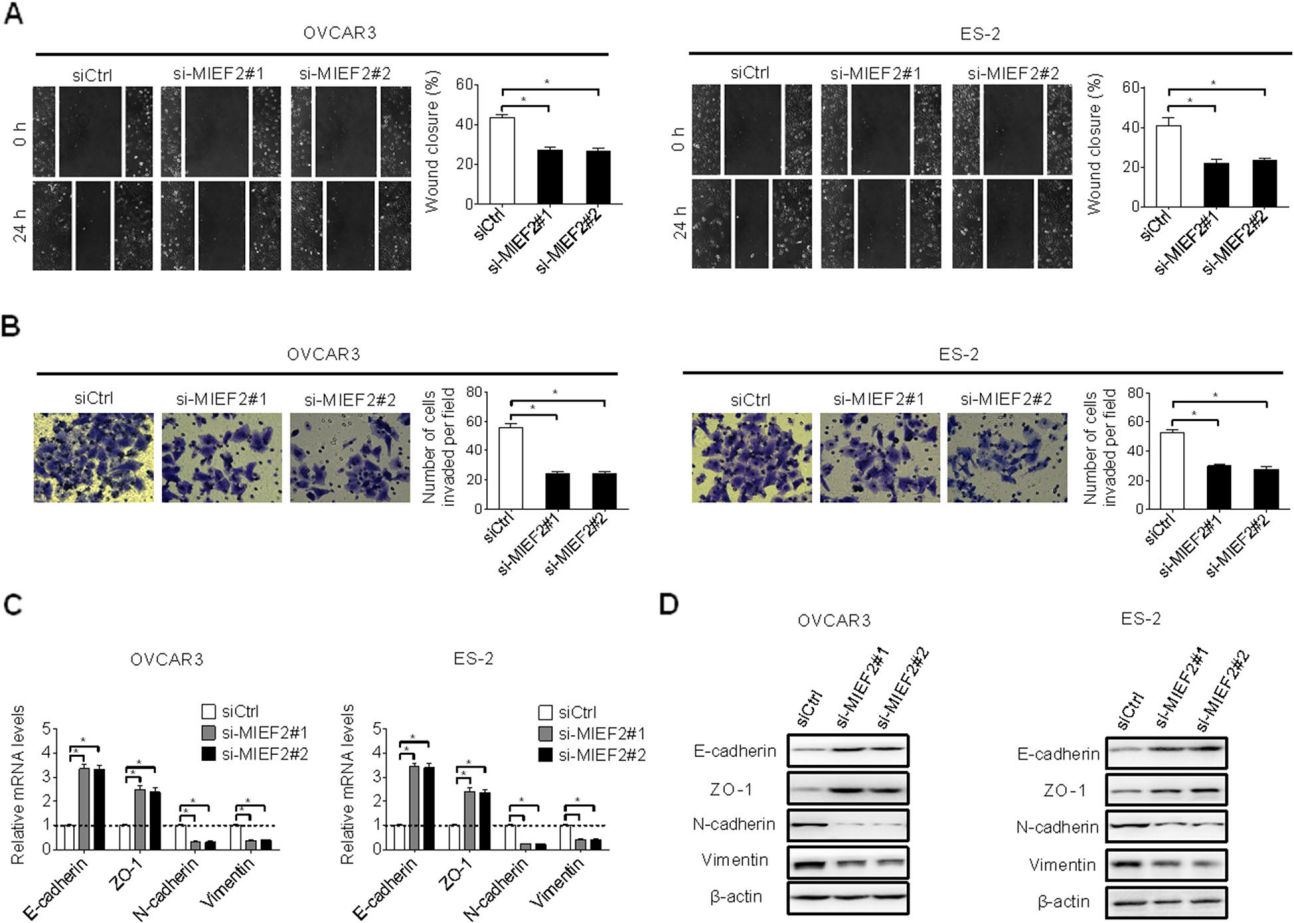
MIEF2 knockdown suppressed migration and invasion of OC cells. **a** and **b** Scratch wound healing and transwell matrigel invasion assays were applied in OVCAR3 and ES2 cells with or without MIEF2 knockdown. siMIEF2, siRNA against MIEF2; siCtrl, control siRNA. **c** and **ds** qRT- PCR and western blot analysis for expressions of epithelial markers of E-cadherin and ZO-1, and mesenchymal markers of N-cadherin and Vimentin in OVCAR3 and ES2 cells with or without MIEF2 knockdown. *P < 0.05

The effects of MIEF2 knockdown on cell migration and invasion of OC cells were also explored. Knockdown of MIEF2 significantly suppressed the migration abilities of OVCAR3 and ES2 cells when compared with control cells (Fig. 3a), as evidenced by wound healing assay. In addition, MIEF2 knockdown also inhibited the invasion abilities of OVCAR3 and ES2 cells, as shown by transwell matrigel invasion assay (Fig. 3b). Previous studies have shown that epithelial-mesenchymal-transition (EMT) plays crucial roles during cancer metastasis through decreasing cell-cell contact and increasing cell migration and invasion [27]. To investigate how MIEF2 controls OC migration and invasion, the expressions of principal epithelial and mesenchymal regulators were determined by qRT-PCR and Western blot analyses. MIEF2 knockdown significantly increased the levels of epithelial regulators of E-cadherin and ZO-1, while decreased the levels of mesenchymal regulators of N-cadherin and Vimentin (Fig. 3c and d), indicating that MIEF2 knockdown suppressed the migration and invasion of OC cells through inhibiting EMT.

### MIEF2 knockdown suppressed OC growth and metastasis in nude mice

**Fig. 4:**
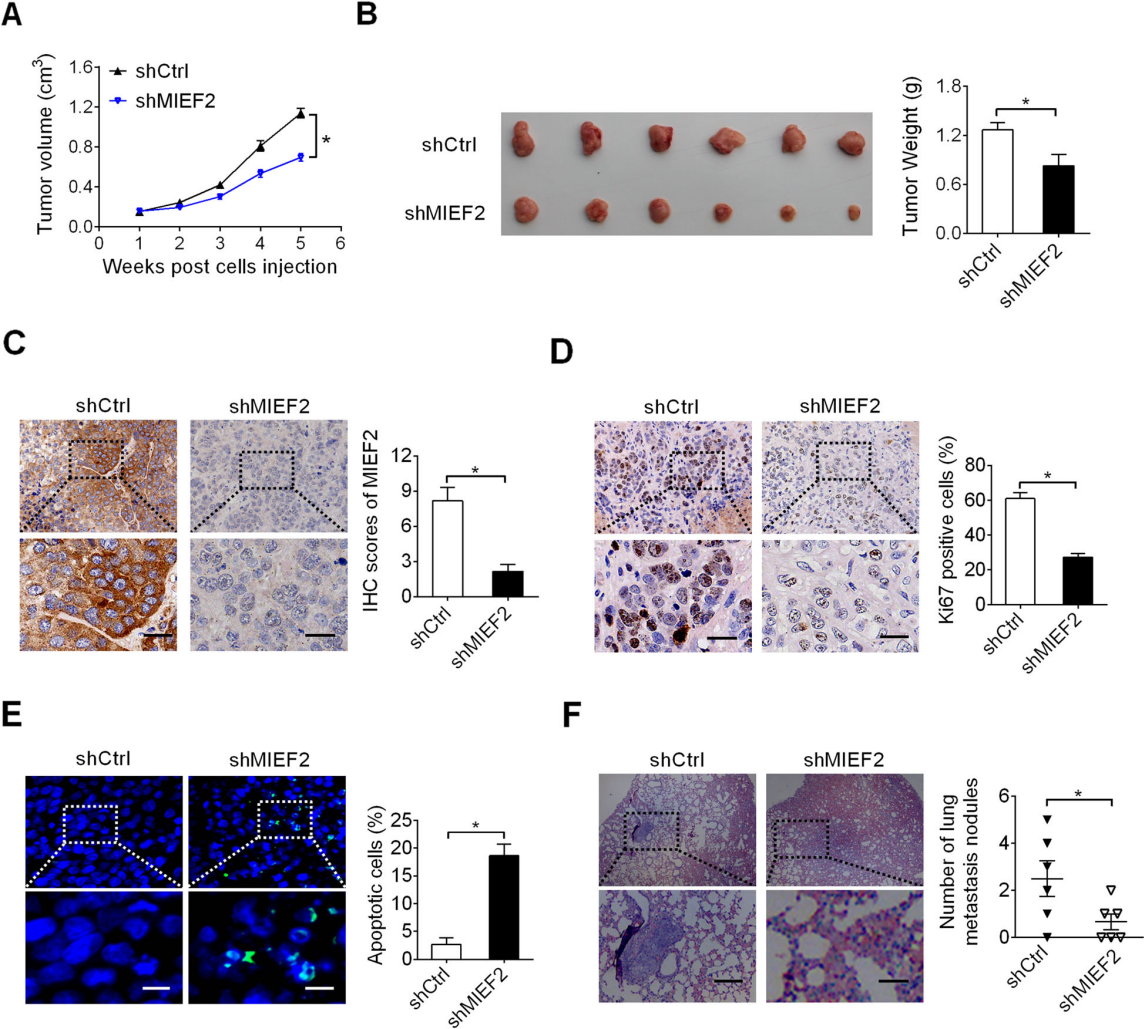
MIEF2 knockdown suppressed OC growth and metastasis in nude mice. **a** and **b** Growth curves and weights of xenograft tumors developed from OVCAR3 cells with or without MIEF2 knockdown. Scale bars, 20 μm. **c** and **d** IHC staining of MIEF2 and Ki-67 in xenograft tumors developed from OVCAR3 cells with or without MIEF2 knockdown. Scale bars, 20 μm. **e** TUNEL assay for cell apoptosis in tumor tissues developed from OVCAR3 cells with or without MIEF2 knockdown. Scale bars, 5 μm. **f** Incidence of lung metastasis of OVCAR3 cells with or without MIEF2 knockdown. Scale bars, 10 μm. *P < 0.05

We then explored the in vivo tumor-promoting effects of MIEF2 in OC. Stably MIEF2 knockdown (shMIEF1) and control (shCtrl) OVCAR3 cells (Fig. S1A and S1B) were injected into the flanks of nude mice to construct xenograft models. MIEF2 knockdown significantly inhibited the growth of tumors (Fig. 4a) and decreased their weights (Fig. 4b). Immunochemistry (IHC) staining showed significantly decreased MIEF2 expression in shMIEF2 tumor tissues compared to shCtrl (Fig. 4c), implying that the tumor growth inhibiting effect was exerted by MIEF2 knockdown. In addition, in line with the in vitro results, significantly fewer proliferating and more apoptotic cells were detected in xenografts from shMIEF2 group compared to those from shCtrl group, as determined by Ki-67 and TUNEL staining assays, respectively (Fig. 4d and e). Moreover, MIEF2 knockdown also significantly suppressed lung metastasis of OVCAR3 cells in nude mice (Fig. 4f).

### Over-expression of MIEF2 enhanced OC cell growth and metastasis

**Fig. 5:**
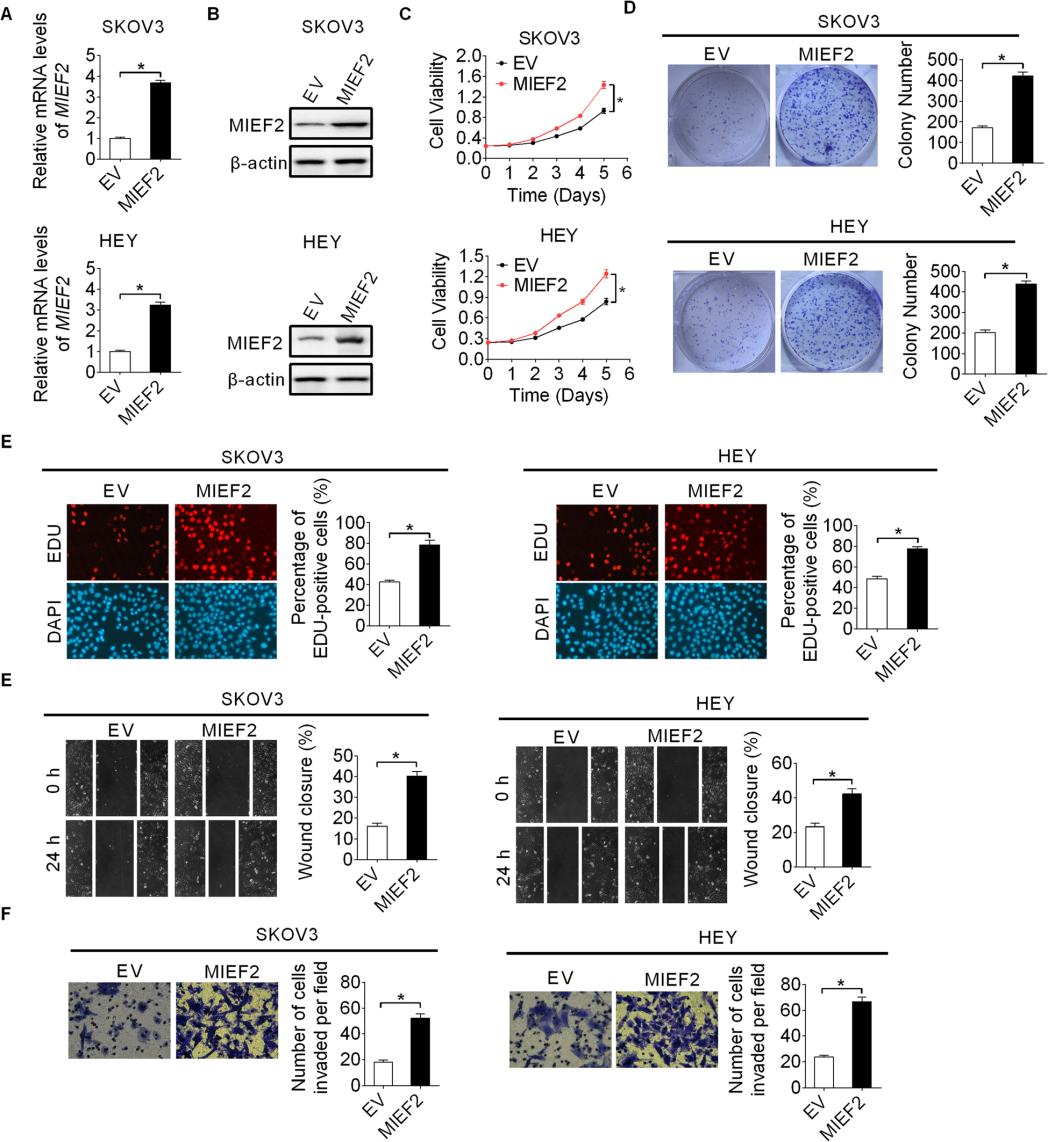
Overexpression of MIEF2 enhanced OC cell growth and metastasis. **a** and **b** qRT-PCR and Western blot analysis for MIEF2 expression in SKOV3 and HEY cells with or without MIEF2 overexpression. MIEF2, MIEF2 expression vector; EV, empty vector. **c** and **d** MTS cell viability and colony formation assays in SKOV3 and HEY cells with or without MIEF2 overexpression. **e** and **f** Wound healing migration and transwell matrigel invasion assays in SKOV3 and HEY cells with or without MIEF2 overexpression. *P < 0.05

To provide further support for the promoting effects of MIEF2 on cell growth and metastasis in OC, MIEF2 was over-expressed in SKOV3 and HEY cells with relatively low MIEF2 expression shown in Fig. 1b and Fig. 1c. Over-expression of MIEF2 expression (Fig. 5a and b) markedly increased the proliferation and colony formation capacities of SKOV3 and HEY cells (Fig. 5c and d). In addition, forced expression of MIEF2 also obviously enhanced the migration and invasion abilities of SKOV3 and HEY cells (Fig. 5e and f).

### MIEF2 over-expression is mainly mediated by the down-regulation of miR-424-5p in OC

**Fig. 6:**
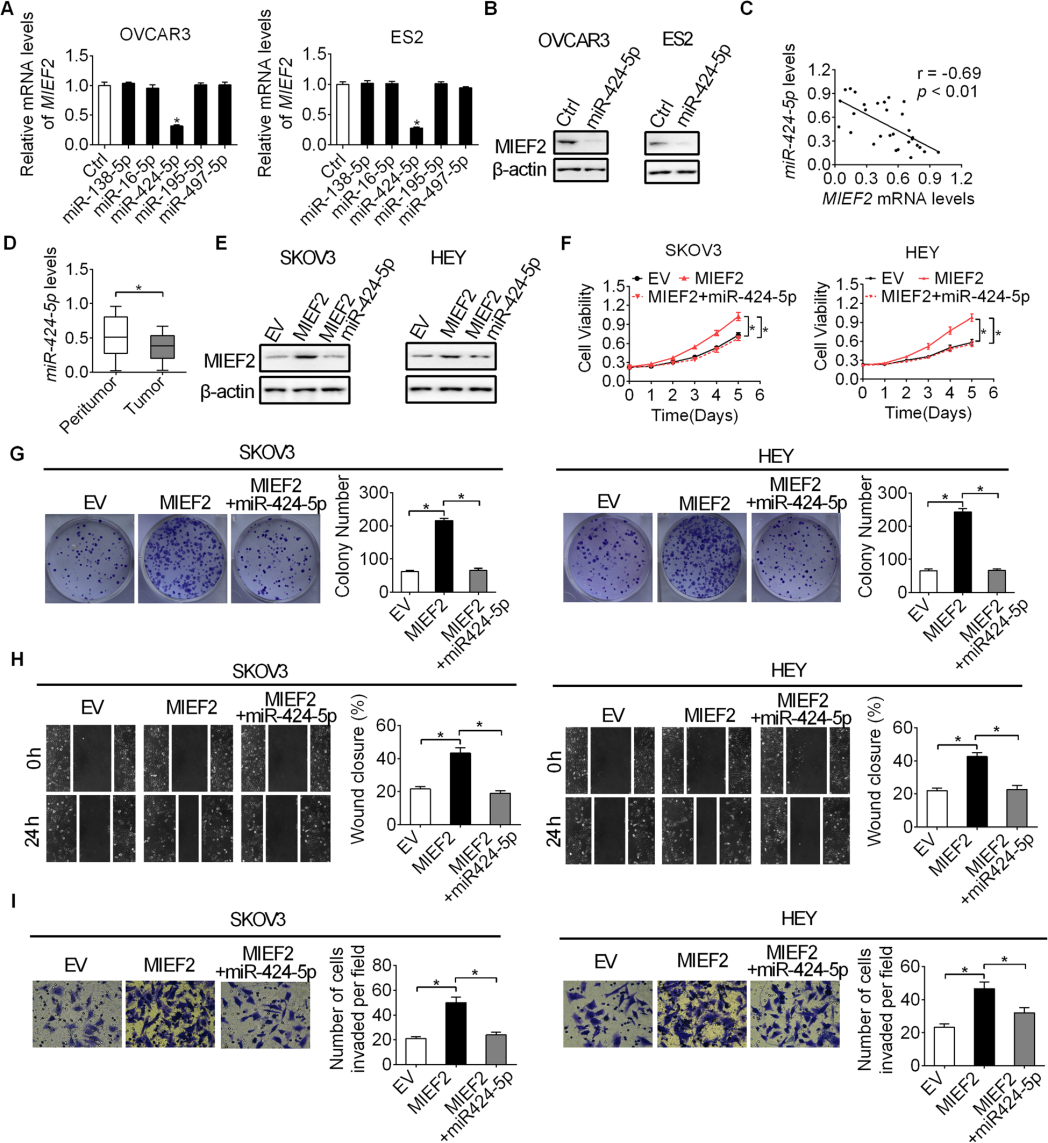
MIEF2 over-expression is mainly mediated by down-regulation of miR-424-5p in OC. **a** and **b** qRT-PCR and Western blot analyses for the expression of MIEF2 in OVCAR3 and ES2 cells after transfection with synthetic miR-424-5p. **c** Correlation between the expressions of MIEF2 and miR-424-5p in tumor tissues from 30 OC patients. **d** The expression of miR-424-5p was determined by qRT-PCR analysis in paired tumor and peritumor tissues from 30 OC patients. **e** The expression of MIEF2 was determined by Western blot analysis in SKOV3 and HEY cells transfected with indicated vectors. **f** and **g** MTS cell viability and colony formation assays in SKOV3 and HEY cells transfected with indicated vectors. **h** and **i** Wound-healing and matrigel invasion assays in SKOV3 and HEY cells transfected with indicated vectors. *P < 0.05

MicroRNAs (miRNAs) are important post-transcriptional regulators of gene expression. To identify potential miRNAs contribute to MIEF2 over-expression in OC, target prediction was applied using microRNA Data Integration Portal (mirDIP) [28]. Among the top five predicted miRNAs targeting MIEF2 (Fig. S2), only miR-424-5p transfection decreased MIEF2 expression in SKOV3 and HEY cells (Fig. 6a and b). In addition, a significant negative correlation was observed between the levels of miR-424-5p and MIEF2 in tumor tissues from 30 OC patients (Fig. 6c). As expected, a significantly down-regulation of miR-424-5p was observed in tumor tissues of OC as compared to their normal counterparts from 30 OC patients (Fig. 6d), indicating that MIEF2 over-expression is mainly mediated by the down-regulation of miR-424-5p in OC. Furthermore, we found that forced expression of miR-424-5p significantly attenuated the promoting effects of MIEF2 over-expression on OC growth and metastasis in SKOV3 and HEY cells (Fig. 6e-i).

### MIEF2 enhanced the Warburg effect in ovarian cancer cells

**Fig. 7:**
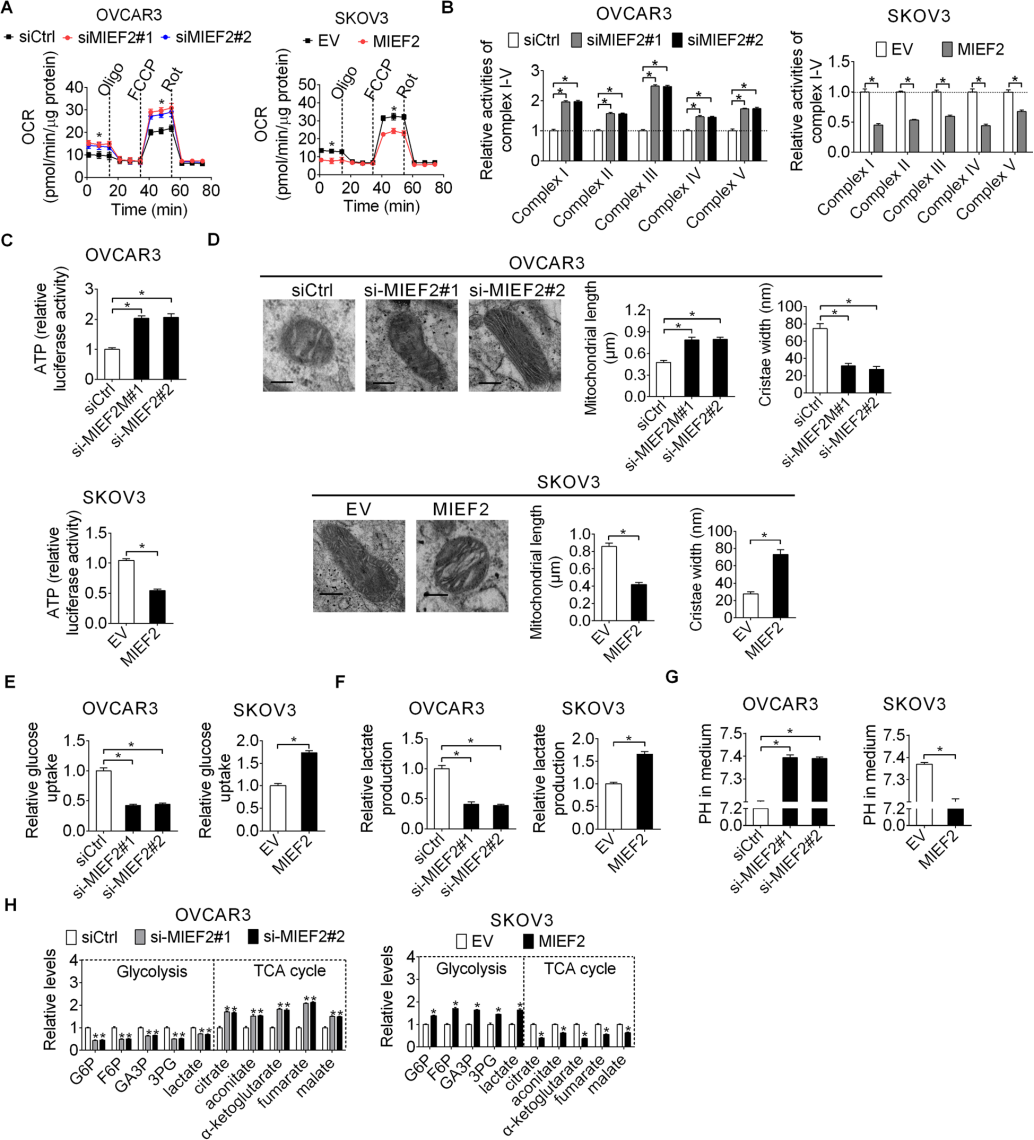
MIEF2 enhanced the Warburg effect in ovarian cancer cells. **a** Oxygen consumption rate (OCR) was determined in OVCAR3 and SKOV3 cells with MIEF2 knocked-down or over-expressed. **b** Relative activities of respiratory complexes I, II, III, IV and V were determined in OVCAR3 and SKOV3 cells with MIEF2 knocked-down or over-expressed. **c** ATP production was measured in OVCAR3 and SKOV3 cells with MIEF2 knocked-down or over-expressed. **d** Representative transmission electron microscopy images of mitochondrial in OC cells with treatment as indicated. Scale bars, 0.2 μm. Mitochondrial length and cristae width were quantitatively analyzed. **e** and **f** Relative glucose consumption and lactate production were determined in OVCAR3 and SKOV3 cells with MIEF2 knocked-down or over-expressed. **g** PH value in cell culture medium was determined in OVCAR3 and SKOV3 cells with MIEF2 knocked-down or over-expressed. **h** Relative intracellular levels of intermediates in glycolysis and TCA cycle were determined by gas chromatography–time of flight–mass spectrometry (GC-MS) analysis in OVCAR3 and SKOV3 cells with MIEF2 knocked-down or over-expressed. *P < 0.05

Mitochondrial plays important roles in the regulation of cellular metabolism. Considering that MIEF2 is a crucial regulator of mitochondrial fission and morphology, we thus hypothesized that MIEF2 over-expression may contribute to the reprogramming of metabolism in OC cells. To define the metabolic alterations induced by MIEF2, we first examined the effects of MIEF2 knockdown and over-expression on mitochondrial oxygen consumption rate (OCR), oxidative phosphorylation (OXPHOS) activity and ATP production. Our results showed that knockdown of MIEF2 in OVCAR3 cells significantly increased the rate of oxygen consumption (Fig. 7a), activities of respiratory chain complexes I-V (Fig. 7b) and ATP production (Fig. 7c), while forced MIEF2 expression exhibited the opposite effects in SKOV3 cells (Fig. 7a-c). Electron microscopy showed that MIEF2 significantly induced mitochondrial fragmentation with increased cristae width (Fig. 7d), a phenotype consistent with mitochondrial OXPHOS defects [29]. These results suggest that MIEF2 suppressed mitochondrial respiration in OC cells mainly through mitochondrial fragmentation-suppressed cristae formation. Considering that impaired mitochondrial OXPHOS is often accompanied by increased glycolysis, which has been well-known as the “Warburg effect”, we accordingly assessed the potential role of MIEF2 in the glycolysis of OC cell. Glucose consumption and lactate production assays revealed that MIEF2 knockdown significantly suppressed glucose consumption and lactate production, whilst pH value in the culture medium was significantly increased. In contrast, MIEF2 over-expression exhibited the opposite effects (Fig. 7e-g). To further corroborate these results, cellular metabolites were relatively quantified by gas chromatography-mass spectrometry (GC-MS) analysis. We found that MIEF2 knockdown resulted in a significant decrease in intracellular concentrations of glycolytic intermediates (glucose 6-phosphate (G6P), fructose 6-phosphate (F6P), glyceraldehyde 3-phosphate (GA3P), 3-phosphoglycerate (3PG) and lactate), while a significant increase in TCA cycle metabolites (citrate, aconitate, α-ketoglutarate, fumarate, malate) in OVCAR3 cells. In contrast, over-expression of MIEF2 was associated with increased glycolytic intermediates, while decreased TCA cycle metabolites in SKOV3 cells (Fig. 7h). These results indicate that MIEF2 switched the glucose metabolism from oxidative phosphorylation to glycolysis in OC cells.

### MIEF2 promoted OC growth and metastasis through activating aerobic glycolysis

**Fig. 8:**
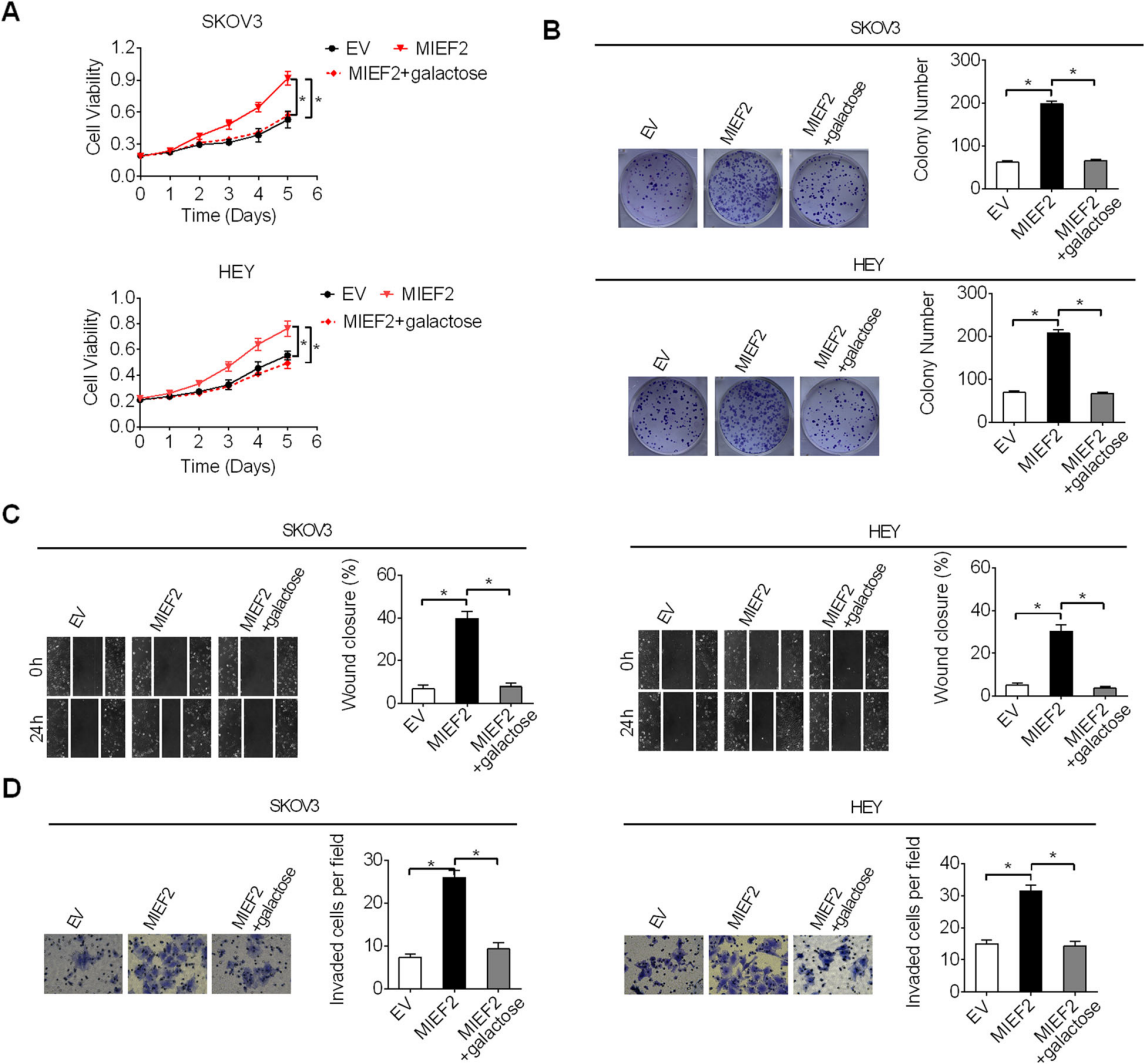
MIEF2 promoted OC growth and metastasis through activating aerobic glycolysis. **a** and **b** MTS cell viability and colony formation assays were performed in SKOV3 and HEY cells treated with or without galactose to suppress glycolysis. **c** and **d** Wound-healing migration and matrigel invasion assays were performed in SKOV3 and HEY cells treated with or without galactose to suppress glycolysis. *P < 0.05

Increased aerobic glycolysis has been coupled with various malignant phenotypes of cancer cells, including tumor growth and metastasis [7, 30]. To test whether the promoting effects of MIEF2 on OC cell growth and metastasis were dependent on increased aerobic glycolysis, glucose in cell culture medium was replaced by galactose (cannot be fermented), which induced cells to rely on mitochondrial metabolism to generate sufficient ATP for survival. As shown in Fig. 8a-d, inhibition of glycolysis by galactose significantly attenuated the growth and metastasis promoted by MIEF2 over-expression in SKOV3 and HEY cells, as determined by MTS cell viability, colony formation, wound healing migration and transwell matrigel invasion assays. These results imply that MIEF2 may exert its oncogenic functions in OC cells through activating aerobic glycolysis.

## Discussion

Mitochondria are the primary energy source for cellular functions, such as cell survival, proliferation, and migration [31, 32]. The morphology of mitochondria is dynamically regulated by the balance between fusion and fission events to maintain energy and metabolic homeostasis [12]. During recent years, a series of studies have revealed the close links between mitochondrial dynamic imbalance and various human cancers [15], including liver [16, 17], breast [18, 19], lung [20, 21], colon [22] and ovarian [23, 24, 33] cancers. MIEF2 (mitochondrial elongation factor 2) is an outer mitochondrial membrane protein involved in the regulation of mitochondrial fission [25]. However, the expression, clinical significance and biological functions of MIEF2 are still largely unclear in human cancers, especially in ovarian cancer (OC). Here, we for the first time demonstrate that MIEF2 is frequently over-expressed in tissues and cell lines of OC mainly due to the down-regulation of miR-424-5p. Over-expression of MIEF2 is associated with poor survival for patients with OC. Consistent with our present findings of MIEF2 in OC, increased expressions of mitochondrial dynamic proteins such as DRP1 (dynamin related protein 1), mitofusin 1 (MFN1) and mitofusin 2 (MFN2) have also been reported in human cancers of liver [16], lung [21, 34], colon [35] and breast [19]. Moreover, significant correlations between the abnormal expressions of mitochondrial dynamic proteins of DRP1 and MFN1 and the prognosis of patients have also been reported in liver [16] and lung cancers. In OC, another critical crucial mitochondrial fission factor MARCH5 has also been reported to substantially up-regulated in tumor tissue in comparison with normal controls [36]. These studies collectively indicate that mitochondrial dynamic dysfunction plays critical roles in the progression of human cancers.

Elevated expression of MIEF2 suggests that MIEF2 may play an oncogenic role in the progression of OC. With this connection, the biological functions of MIEF2 were explored both in vitro and in vivo. We found that MIEF2 knockdown markedly suppressed the viability and colony formation abilities of OVCAR3 and ES2 cells, while forced expression of MIEF2 significantly increased the viability and colony formation abilities of SKOV3 and HEY cells. Subcutaneous tumor models further confirmed that knockdown of MIEF2 significantly attenuated the growth abilities of OC cells in nude mice. Similarly, over-expression of another mitochondrial fission factor DRP1 has also been shown to promote tumor cell growth in human cancers of liver [16], lung [21] and breast [37]. Given that cell growth is determined by both cell proliferation and apoptosis, we thus explored the mechanism by which MIEF2 knockdown suppressed OC cell growth and found that MIEF2 knockdown suppressed OC cell growth through both inducing G1–S cell cycle arrest and cell apoptosis. In line with this, Ki-67 and TUNEL staining assays also demonstrated fewer proliferating cells and more apoptotic cells in MIEF2 knockdown subcutaneous tumors compared to the controls.

In addition to tumor growth, the role of MIEF2 in the metastasis of OC cells was also investigated. Knockdown of MIEF2 in OVCAR3 and ES2 cells significantly suppressed their migration and invasion abilities. Conversely, overexpression of MIEF2 enhanced the migration and invasion abilities in SKOV3 and HEY cells. Consistently, increased expression of another mitochondrial fission regulator MARCH5 has also been reported to promote the migration and invasion of OC cells both in vitro and in vivo [36]*.* Moreover, we found that MIEF2 exerts its metastatic promoting role in OC through inducing epithelial-mesenchymal transition (EMT). Similarly, a previous study in hepatocellular carcinoma has also indicated that silencing of another mitochondrial fission protein MTP18 markedly suppressed the invasion abilities of pancreatic cancer cells through inhibiting EMT [38]. By contrast, as a mitochondrial fusion protein, MFN1 has been shown to play an EMT suppressive role in HCC [39]. These observations collectively indicate that dysregulated mitochondrial dynamics play crucial roles during epithelial-mesenchymal transition and metastasis in cancer cells.

MicroRNAs (miRNAs) are important post-transcriptional regulators of gene expression. miR-424-5p has been established as a novel tumor suppressor that was frequently down-regulated in several types of cancer, including breast cancer [40], hepatocellular carcinoma [41], bladder cancer [42] and cervical cancer [43]. A previous study in ovarian cancer also has reported that miR-424-5p was significantly down-regulated and promoted cell proliferation [44]. Consistently, our present study also revealed a significant down-regulation of miR-424-5p in OC cells. Furthermore, we demonstrated that the down-regulation of miR-424-5p contributed to MIEF2 up-regulation and thus tumor growth and metastasis in OC. However, we still cannot rule out the possibility that other genetic or epigenetic alterations may also contribute to the overexpression of MIEF2 in OC.

Reprogrammed glucose metabolism characterized by preferential dependence on glycolysis versus oxidative phosphorylation (OXPHOS) for energy production (also known as Warburg effect), even in the presence of oxygen, has been known as a hallmark of cancer [4]. Although several oncogenes such as myc and RAS have been shown to play important roles in this metabolic reprogramming [45], the key plays contribute to increased aerobic glycolysis in cancer cells still needs further investigation. Glucose metabolism in cancer is balanced by glycolysis and mitochondrial OXPHOS [46]. During the past several decades, mitochondrial malfunction has been revealed as one of the most common reasons for increased aerobic glycolysis in cancer cells [10, 21]. However, identification of novel regulators contributing mitochondrial dysfunction and thus increased aerobic glycolysis is still urgently needed. Here, we revealed that over-expression of MIEF2 significantly promoted the metabolic switch from oxidative phosphorylation to glycolysis in OC cells. Moreover, we found that enhanced aerobic glycolysis was involved in MIEF2-promoted tumor growth and metastasis. These results suggest that mitochondrial dysfunction plays a crucial role in the reprogramming of glucose metabolism and thus tumor progression in human cancers.

## Conclusions

In summary, we show for the first time that MIEF2 is commonly over-expressed in OC and its over-expression is associated with poor survival for patients with OC. MIEF2 plays a crucial oncogenic role in the progression of OC through reprogramming glucose metabolism from oxidative phosphorylation to glycolysis. Our results suggest MIEF2 as a novel prognostic marker and therapeutic target in treatment of OC.

## Supplementary Information

**Additional file 1.**

## Data Availability

The datasets used and/or analyzed during the current study are available from the corresponding author on reasonable request.

## Abbreviations

OC: Ovarian cancer
MIEF2: Mitochondrial elongation factor 2
qRT-PCR: Quantitative real-time PCR
IHC: Immunohistochemistry
DRP1: Dynamin-related protein 1
MFN1: Mitofusion 1
siRNA: Small interference RNA
PVDF: Polyvinylidene fluoride
H&E: Hematoxylin and eosin
OCR: Oxygen consumption rate
OXPHOS: Oxidative phosphorylation
ATP: Adenosine triphosphate
GC-MS: Gas chromatography-mass spectrometry
TCA cycle: Tricarboxylic acid cycle
TUNEL: Terminal deoxynucleotidyl transferase-mediated dUTP-biotin nick end labeling
EMT: Epithelial-mesenchymal transition
