# Supplementary material for: MIEF2 over-expression promotes tumor growth and metastasis through reprogramming of glucose metabolism in ovarian cancer
Source: J Exp Clin Cancer Res. 2020 Dec 14;39:286. doi: 10.1186/s13046-020-01802-9 (PMC7737286; doi:10.1186/s13046-020-01802-9)
Supplement: Supplementary file 1 — Additional file 1. [file 13046_2020_1802_MOESM1_ESM.docx]

**Supplemental information**

**MIEF2 over-expression promotes tumor growth and metastasis through reprogramming of glucose metabolism in ovarian cancer**

**Supplemental figures**

**
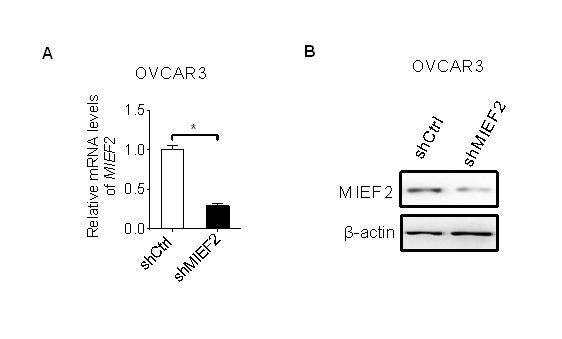
**

**Figure S1.** Stable knockdown of MIEF2 in OVCAR3 cells was determined by qRT-PCR **(A)** and Western blot (**B**) analysis.

**
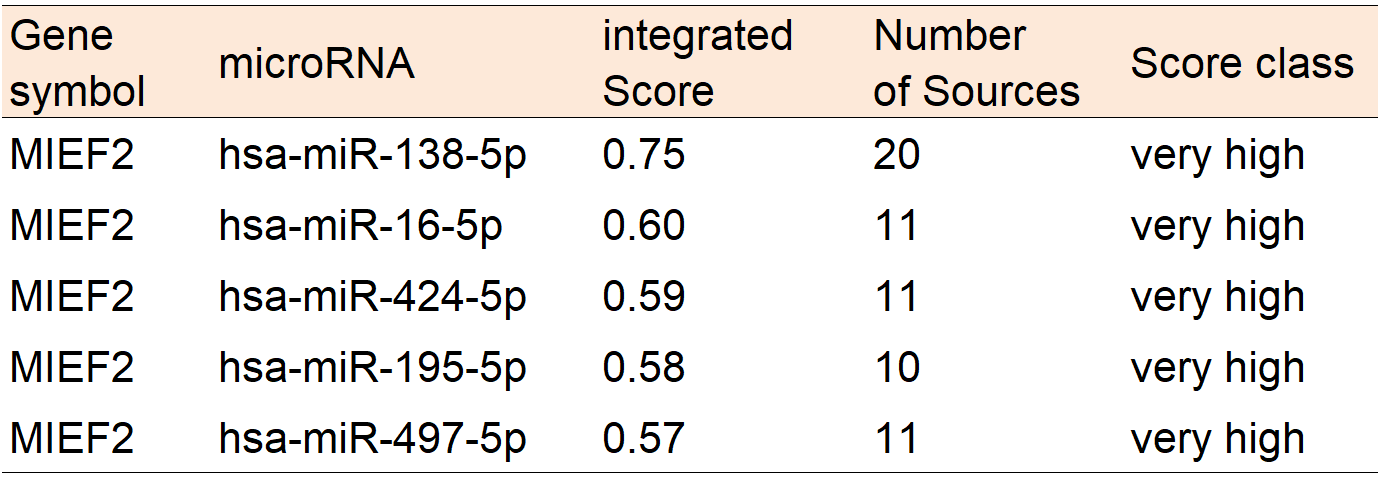
**

**Figure S2.** Top five predicted miRNAs targeting MIEF2 using the microRNA Data Integration Portal (mirDIP)-based target prediction^[1]^.

**Supplementary Tables**

**Table 1.** Sequence of primers for qRT-PCR analysis

| *MIEF2* | forward primer | CAGAAACGGGGGAAGCGG |
| --- | --- | --- |
|  | reverse primer | CACCAGGAGACGCACATGG |
| *E-cadherin* | forward primer | AAAGGCCCATTTCCTAAAAACCT |
|  | reverse primer | TGCGTTCTCTATCCAGAGGCT |
| *Z0-1* | forward primer | CGACCAGATCCTCAGGGTAA |
|  | reverse primer | TCCATAGGGAGATTCCTTCTCA |
| *N-cadherin* | forward primer | AGCTCCATTCCGACTTAGACA |
|  | reverse primer | CAGCCTGAGCACGAAGAGTG |
| *Vimentin* | forward primer | GACGCCATCAACACCGAGTT |
|  | reverse primer | CTTTGTCGTTGGTTAGCTGGT |
| miR-424-5p | forward primer | TTTATTCACCCGCAGGTACCCC |
|  | reverse primer | GCAGACCCCACCTTCTACCT |
| U6 | forward primer | CTCGCTTCGGCAGCACA |
|  | reverse primer | AACGCTTCACGAATTTGCGT |
| *β-actin* | forward primer | AGGCACCAGGGCGTGAT |
|  | reverse primer | GCCCACATAGGAATCCTTCTGAC |

**Table 2.** Primary antibodies used in this study.

| **Antibody** | **Company (Cat. No.)** | **Working dilutions** |
| --- | --- | --- |
| MIEF2 | abcam (ab101350)  abcam (ab247033) | WB: 1/1000; IHC:1/200 |
| E-cadherin | abcam (ab1416) | WB: 1/1000 |
| Z0-1 | abcam (ab190085) | WB: 1/1000 |
| N-cadherin | abcam (ab98952) | WB: 1/1000 |
| Vimentin | abcam (ab8978) | WB: 1/1000 |
| Ki-67 | Proteintech (27309-1-AP) | IHC:1/300 |
| β-actin | Proteintech (20536-1-AP) | WB: 1/1000 |

**Reference**

[1] Tokar T, Pastrello C, Rossos AEM, et al. Mirdip 4.1-integrative database of human microrna target predictions [J]. Nucleic Acids Res, 2018,46(D1):D360-D370.
